# Equality and poverty: views from managers and professionals from public services and household heads in the Belo Horizonte Metropolitan Area, Brazil

**DOI:** 10.1186/s12939-020-01243-y

**Published:** 2020-08-06

**Authors:** Viviane Helena de França, Celina Maria Modena, Ulisses Eugenio Cavalcanti Confalonieri

**Affiliations:** 1 Departamento de Medicina, Campus Avançado de Governador Valadares, Universidade Federal de Juiz de Fora, Rua Manoel Byrro, 241 – Vila Bretas, Governador Valadares, Minas Gerais CEP: 35032-620 Brazil; 2 Instituto René Rachou, Fundação Oswaldo Cruz Minas, Avenida Augusto de Lima, 1715, Bairro Barro Preto, Belo Horizonte, Minas Gerais CEP: 30190-009 Brazil

**Keywords:** Quality of life, Public policies, Health promotion, Intersectoral action, Management, Health equity

## Abstract

**Background:**

Tackling poverty requires reconsideration of quantitative factors related to “who” is poor and by “how much” and qualitative factors addressing “what poverty means in these individuals’ lives”. Greater understanding is required concerning the types of access actually used by families in poverty in attempts to meet their basic needs. Poverty must be addressed based on the question: “Inequality of what?” It is in reflecting on the realities of such groups when their basic needs are not met that public policies can be improved and implemented with legitimate priorities. Objective: Describe coverage and access to public health, education and social assistance services and the related effects on the quality of life of families in extreme poverty.

**Methods:**

An exploratory mixed methods study was conducted applying Amartya Sen’s “Basic Capability Equality” framework, with: 1) 27 interviews with managers and professionals from public services serving territories with extreme poverty; 2) Survey with a systematic proportionate stratified sample of 336 heads of households in extreme poverty from a total 2605 families. The resulting data was analyzed with thematic content analysis and descriptive statistics, respectively.

**Results:**

The managers and professionals described the lives of families in extreme poverty with phrases such as, “*These people suffer. Sadness weighs on their lives!*” and “*Depression is the most common illness*”. Their precarious circumstances and inadequate access were cited as causes. Quality of life was considered bad or very bad by 41.4% of heads of households. A total income of less than one-third of the minimum wage was received by 56.9% of the sample. One or more people were unemployed in the family in 55.8% of cases. For 53.3% of heads of households, public services “*did not meet any or few of their needs”.* The main social determinants of health were described as: alcohol and drugs (68.8%); lack of good health care (60.7%); and absence of income/work (37.5%). The following were identified as solutions to improve their quality of life: (1) health (40.5%); (2) education (37.8%); and (3) employment (44.6%).

**Conclusions:**

The social determinants of poverty and health must be addressed jointly through intersectoral public policies and egalitarian mechanisms that promote investment in social protection.

## Background

According to data from 2018, 3.4 [1] billion people around the world live in poverty while 736 million live in extreme poverty, with less than $1.90 per day [2]. A 2019 study on the Multidimensional Poverty Index, monitoring 101 countries and 5.7 billion people, observed that 1.3 billion people worldwide (23.1% of the population) are multidimensionally poor in terms of health, education, and standard of living [3]. In 2018, while the worldwide rate of extreme poverty was the lowest ever (8.6%)—indicating enormous progress in tackling this challenge—difficulties persist in countries dealing with conflict and political instability, as evidenced by their consistently high percentages of this indicator. From 1990 to 2015, the percentage of the world’s population living in extreme poverty dropped from 36 to 10%. But there are still challenging regions, such as in sub-Saharan Africa where 40% of the population is extremely poor, and Latin America where this figure is 30.2% [2]. In Latin America, poverty and extreme poverty continue to constitute alarming problems as 185 million people live in poverty and 66 million lived in extreme poverty [4]. In Brazil, a comparison focusing on extreme poverty between 2012 and 2014 showed that there was a major reduction in poverty, reaching as low as 4.5% of the population. However, since 2015, with the socioeconomic crisis that has since gripped the country, this contingent has increased [5]. Data from 2018 highlights that 20.1% of Brazil’s population lived in poverty, with $5.50/day (US), while 6.5% lived in extreme poverty with incomes of less than $1.90 per day (US) [6].

Tackling poverty requires reconsideration of not only quantitative factors related to “who” is poor and by “how much” they are poor, but also qualitative factors addressing what the situation of poverty means in the lives of these individuals. It is necessary to reflect on the types of access actually used by families in extreme poverty as they seek to meet their basic needs [7–9]. Indeed, it is imperative to gain understanding of the phenomenon of poverty based on the question: Inequality of what? [9] What is referred to in this question is the paramount importance of not only quantifying aspects of inequality within the population, such as income, education, health and housing, but mainly of gaining understanding of “how” and “why” such disparities work in a certain way. Why they involve contexts marked by prejudice and/or losses in quality of life [8]. The current debate on the concept of poverty should be based on more than income deprivation. It must also address deprivation of access to the items necessary to live a dignified life, which include aspects such as freedom, education, health, rights, employment and quality of life. It must consider the ensuing realities when the basic needs of these groups are not met. When the ongoing debates adequately do so, it will provide guidance to establish more appropriate priorities for the implementation of public policies [8].

In general terms, understanding poverty in an expanded way requires analyses of circumstances emphasizing the “idea of deprivation” or access. The definition of access can be understood as consisting of four elements: namely availability, acceptability, affordability, and information. Availability consists in the “existence or non-existence of the service in the appropriate place and at the time it is required”. In this way, it broadly encompasses the geographical and physical relationship of institutions in relation to the individual’s need [9]. Acceptability reflects the population’s perception of services. Affordability refers to the price of services and individuals’ purchasing power, including the possibility of paying for the direct or indirect costs of service provision. Information refers to the knowledge that allows the services to actually be used. Information is required in adequate quality and quantity for people to be able to enjoy the service network and use it sufficiently and adequately in a way that allows them to resolutely fulfill their basic needs [9].

The desires and choices of people in situations of poverty suggest the prevalence of a series of integrated factors. Taken together, these factors constitute a process of denial of the right to social justice. Based on that, the right to fulfillment of such individuals’ basic family needs is also denied and access to goods and services are persistently lacking. These circumstances ultimately cause processes marked by situations of extreme poverty. A repetitive cycle of constraints continuously assails these families, further accentuating their social exclusion in what can be referred to as the “inter-generational poverty transmission cycle” [10]. Such constraints include low income and a stunted ability to acquire essential goods and services required for a dignified life. Those constraints have an adverse impact on: levels of health and education; access to treated water and sanitation services; physical safety; autonomy and an active voice in society; and opportunities for economic growth, social inclusion and human capital development [11]. In reference to health, the precarious living conditions that prevail in contexts of extreme poverty are also reflective of several detriments to the world’s population. Persons in extreme poverty are affected by a prevalence of higher mortality and failures or absences in prenatal care and teenage pregnancy. The latter is due to early marital and reproductive relationships that result in parents taking on responsibilities without being adequately mature to deal with the related requirements. Consequently, a chain of factors ensues that may lead to worsened living conditions. In sum, the living standards of those living in poverty throughout the world do not reflect their legitimate desires and choices [12].

To act on these factors and provide improvements to the quality of life of families in extreme poverty, collective decision-making in the process of public policy implementation is required. Moreover, the various service providing sectors must be involved in such processes in concert, since health services or other isolated sectors cannot act on such macro determinants alone. In this regard, the World Health Organization has proposed a global movement with the creation of several commissions and initiatives--worldwide and by nation. Their objective is to address issues that are implicit to social determinants, including the social determinants of health together with those of poverty [13, 14]. Such social determinants include individuals’ experiences in their first years of life and within the family, access to education, the stability of their economic situation, employment and decent work, and housing to name but a few. These determinants also consider how influences from the community, regional, national, and global spheres contribute to harming the environment, global health, and generating unfair, unequal economic, social and cultural systems. After all, the results of such determinants lead—to a greater or lesser extent—to gains or losses for human value, basic rights, citizenship and dignity in the face of situations of social injustice. They entail “worsened living conditions and the breakdown of universal health and social protection services”, contributing to poverty and extreme poverty and increased detriments to public health [13, 14]. In this regard, in several cases, country members of the Organization of the Petroleum Exporting Countries (OPEC) have validated that investments in developing human capital, such as in health and education, can significantly reduce poverty while increasing economic growth [15].

In 2015, 193 State members of the United Nations came together to establish the 2030 Sustainable Development Agenda. An established principle of the Agenda is to advance equality and Sustainable Development Goal (SDG) 1 aims to “end poverty in all its forms everywhere”. The implementation of social protection systems, among other measures, are recommended as a way of attaining the objectives of this and the other 16 SDGs. Such systems would contribute positively to achieving equality through health, ensuring a healthy life and promoting well-being for people of all ages (with universal health coverage), education, societies with inclusive economic growth, employment and decent work, and more [16, 17]. The United Nations Development Programme (UNDP) emphasizes that the capabilities approach, as posited by Amartya Sen, is a poverty assessment method that defines poverty, not merely as a matter of actual income, but as an inability to acquire certain minimum capabilities [18]. In this regard, monitoring health inequalities among individuals living in situations of poverty can provide valuable evidence to inform public policies, programs, and practices on how to address equality in alignment with the intersectoral nature of the SDGs. Indeed, the multiple dimensions of inequality may broadly have an impact on social, economic, demographic and other health-related aspects across a society’s population [16].

Understanding the different dimensions of inequality affecting families in poverty requires different assessment methods to appreciate the context of their lives. Such methods may reflect the reality they face in objective or subjective terms, but can be used to complement the implementation of legitimate public policies in any case [19]. Persons living in poverty are excluded in this world and their experiences have been impacted upon negatively—one must not forget to appreciate this. Indeed, to achieve a comprehensive understanding of human poverty, such persons must be given opportunities to express their perception of their own survival [18].

This study used a mixed methods (qualitative and quantitative) approach, based on the “Basic Capability Equality” theoretical framework set out by Amartya Sen [9], to conduct exploratory research. This framework was adopted as a principle of justice based on the rights to life and health, citizenship, and well-being and was addressed by analyzing the realities of families living in extreme poverty, their quality of life and access to public services, and the social determinants of their health and their situation of poverty. Thus, the study had two main objectives. The first objective was to understand the views of managers and professionals of public services—via semi-structured interviews—regarding public policies, coverage of and access to public services for health, social assistance and education, and their effects on the quality of life and health of families living in extreme poverty in a municipality with high social vulnerability in the Belo Horizonte Metropolitan Area (BHMA). The second objective was to understand the views of heads of households with a profile of extreme poverty and the target audience of the *Bolsa Família* (Family Allowance) Program (BFP)—via a survey—in the same municipality of the BHMA, regarding local quality of life, coverage of and access to public policies and health services, social assistance, education, and the effects thereof on their quality of life.

## Theoretical framework

In the present study, poverty was addressed as a multidimensional concept involving situations and bonds marked by contexts of social inequality. Public policies of the social, economic and environmental spheres exist in these spaces, each with their effects on the territories where poor families live and co-exist. Such policies produce disadvantageous situations insofar as the role of universal rights differs among them, including coverage of and access to public services; consumer goods and other resources available in the community to meet residents’ basic needs; and opportunities to develop residents’ personal potential, autonomy and independence, and achieve overall well-being.

Amartya Sen’s “Basic Capability Equality” [9] (BCE) framework was employed as the theoretical framework for this study, since it represents “the concept of distributive justice theory that best translates the concerns of those with strong egalitarian sentiments”. This theory embodies a relevant debate on the contemporary normative political theory that took shape following John Rawls’ “Theory of Justice”, from 1971, concerning equity. Amartya Sen addressed the social equity-based concepts related to distributive justice in 1979, rejecting a utilitarian position, which Rawls also rejected.

For Rawls, issues related to distributive equality focus on the means to ensure freedom; however, for Sen, equality should be emphasized from the notion of freedom itself. The latter’s understanding is that equality as a commodity or a means to ensure freedom should eliminate all “commodity fetishism” [9, 20]. Rawls proposes a focus on original equality based on the equal distribution of primary goods, while Sen does not address equality with an emphasis on such primary goods themselves. This is his stance since he considers these goods to be incomplete and displaced from reality when taken for an individual. In this way, they would be insufficient to provide what people actually need and long for as life values [9, 20, 21]. In this debate between Rawls and Sen, the latter proposed the theory of BCE [9] as a notion of distributive justice beyond the principles of “pure procedural justice”. He demonstrated that interpersonal advantages open up significant spaces in individuals’ lives endowed with a sort of citizen’s standard. In other words, he highlighted a type of well-ordered social cooperation that does not actually leave room for everyone to develop their basic capabilities at minimal levels of decency. In order to accomplish this, people’s “capability to function” as active and cooperative members of society must reach certain distributive thresholds. Once such thresholds have been met, people would experience inclusion not only based on improved access to income but, particularly, through enhanced opportunities for self-realization, leisure, and enjoyment of their families and children [9].

According to Sen, promoting social equity—from the perspective of BCE development—requires primary goods to be available to families, to help them to meet their basic needs and not hinder or obstruct their development and self-realization as concerns freedom of choice; in other words, their potential. Thus, public service provision and types of access that sustain families’ quality of life in the territories where residents’ daily lives and coexistence take place must be considered in a systematic way. Means and ends must be provided together in order for individuals to develop their capabilities and carry out relevant activities, which is also referred to as functionings. With adequate functionings, other prospects can be generated for the lives of families in poverty, thereby providing them with better opportunities of choice [9].

Therefore, providing for the essential needs of such families requires viable public policies—in formal and practical terms—that decant down to local contexts. The related services should enable easy and consistent access to residents of territories with extreme poverty [22]. Thus, this study focuses on BCE as a method to obtain a broader understanding of contexts of extreme poverty. It legitimizes the process of listening to families living in extreme poverty speak about their main problems from their point of view regarding material deprivations (objective data such as income, education, access to drinking water and sanitation, housing, health, employment, transportation, among others) and subjective matters (including self-realization, autonomy, satisfaction with quality of life, a healthy life). All of these aspects are valuable in the pursuit of a dignified life, a sense of citizenship and overall well-being.

## Methods

### Study location

The study took place in Ribeirão das Neves (RN), one of the 37 municipalities in the Belo Horizonte Metropolitan Area (BHMA), Minas Gerais, in the South-Eastern region of Brazil. The BHMA is home to 4.8 million inhabitants, of which 1.4 million live in poverty [23]. Belo Horizonte (BH) has an estimated population of 2,375,151 people [24] and an incidence of poverty of 5.43% [25]. RN is about 40 km away from BH (the city) and encompasses an area of 154.18 km [2]. It is intimately integrated with the capital of BH owing to the large contingent of workers that flow to this area in search of employment and income. RN has 334,858 inhabitants [26], a high concentration of low-income households, and an incidence of poverty of 13% [26]. It lacks an economic base with which to absorb part of its workforce, which is classified as “popular”. RN has historically taken shape as a biproduct of the disorderly growth experienced in the capital of the BHMA. Expansion from the center to peripheral territories took place due to the limited capacity of Belo Horizonte to accommodate the high number of families migrating to the region as early as 1950. RN started as an urban patch with the characteristics of an agglomeration and a precarious spatial occupation [27, 28]. Resources and investments fail to meet the demand for public services and infrastructure, thus reinforcing social exclusion and spatial segregation within its population. Moreover, these dynamics coexist with the fact that RN is host to a large prison complex composed of five institutions that contribute to the prejudice associated with the municipality and its local population [27, 28].

### Mixed methods

This is an exploratory study applying a mixed-method (qualitative-quantitative) methodological approach, as defined by Creswell and Clark (2013) [29]. Initial data collection and data analysis were performed in separate, sequential steps. Subsequently, in the discussion, the results of those steps are introduced to provide an in-depth understanding of the findings.

First, qualitative data was collected, analyzed and used in an intermediate phase to develop the quantitative data collection phase. That intermediate phase was used as an opportunity to plan a rigorous assessment instrument with adequate psychometric properties. In exploratory research projects, the subjects chosen to participate in the qualitative and quantitative phases are not the same since the purpose is to generalize the results for a given population. Thus, the participants from the quantitative phase are different than those in the qualitative phase, allowing for a greater sample with which to draw general conclusions about the studied phenomenon [29].

This study encompassed four phases, the characteristics of which are described below. These phases sought to answer the following questions:

- What is the quality of life of families living in extreme poverty?
- What are their coverage levels and access to public services as it relates to what is needed to meet their essential everyday needs, develop their human potential, and provide them well-being?
- What are the main social determinants of poverty affecting such families and what are the associations with the social determinants of health?
- What programming can be suggested via investments in public policies to improve their quality of life?

#### Phase 1 – Qualitative

Semi-structured qualitative interviews were carried out with 27 managers and professionals (M/Ps) of the departments of Municipal Health (MHD), Social Assistance (SAD) and Education (DE). The study sought to investigate their views of public policies, coverage and access provided by the public health, social assistance and educational services of RN. It also sought their views on the related effects on the health and quality of life of families in extreme poverty. The criteria for their inclusion in the study were the following:

- M/Ps responsible for tasks related to the planning and execution of public service programming in the fields of health, education or social assistance, including the *Bolsa Família* (Family Allowance) Program (BFP);
- M/Ps carrying out the tasks related to the aforementioned public policy fields in the service of the RN population living in extreme poverty during the preceding 6 months, at least.

The researchers personally invited the interviewees to take part in the study. Subsequent interviewees were sought out as per the “snowball sampling” technique, following the sequential recommendations of M/Ps at the central, regional, and local level who held leadership positions in the municipal administration. The number of interviewees was determined by the concept of theoretical saturation. This technique can be applied when qualitative samples are made up of a small number of subjects representing a certain subpopulation and the final number of interviewees is determined in the field. Ending the data collection phase is justified once the information gathered starts to become redundant and/or repetitive, not representing any contribution of new ideas [30, 31].

A semi-structured interview guide was created and validated (tested in another field) previously. This script was designed with a basis in the work of Minayo (2014) [32] and encompassed two parts addressing the following questions:

- The socio-demographic profile of the M/Ps of the public services; age; level of education; profession and institution of employment; service time and employment type; role occupied in the respective Municipal Department of Health, Social Assistance or Education.
- Quality of life of the families living in extreme poverty in RN: What is your perception of the quality of life of the population in RN that is socially vulnerable or in extreme poverty? How would you describe the regions and micro-regions of RN where the majority of the population are living in situations of extreme poverty and social vulnerability? What are the main problems experienced by the socially vulnerable population in terms of quality of life? Why? (Associating quality of life with matters related to health in the urban sphere). What have the public authorities done in this region to improve the living conditions of this population? Explain the public policies, programs, and current activities in RN. Has the quality of life of the population who is socially vulnerable or living in extreme poverty changed since such public policies and programs have been implemented? In what way?

Interviews were conducted individually and lasted 90 min on average. Data collection took place between December 2013 and February 2014 with audio-recorded files. The data analysis process was carried out manually; the interviews were transcribed, categorized and analyzed using Bardin’s (2001) [33] thematic content analysis technique. This content analysis technique is considered a systematic and objective procedure to generally organize information and categorize the main ideas identified in the data sets. Thus, the technique enables demarcation of themes, subthemes, and subsequently of entire thematic axes, based on interviewees’ accounts. In sum, this process makes it possible to group themes and subthemes in relation to a central idea through significant qualitative indicators that address the study’s questions [33].

#### Phase 2 – Intermediate

Construction of the structured survey instrument, based on the analysis of the data from the first stage. The BCE [6] framework was adopted to investigate the views of heads of households in extreme poverty (residents of RN) regarding the quality of local life, current public policies, coverage, access to public services, and health measures and effects on their quality of life. Incorporating the BCE framework allowed questions to be included in the survey addressing the notion of equity in terms of public service coverage and access thereto by families. It also justified the inclusion of questions on how the previous issues affected individuals’ development of basic capabilities, human capital, and overall well-being. The following theoretical principles and constructs were applied to the survey to obtain an appreciation for both the objective and subjective aspects of the quality of life of families in extreme poverty:

- Broadened concept of quality of life, integrating human development and the right to life, the sentiment of citizenship, autonomy, self-realization and all members of a nuclear family exploiting their own potential.
- The central family sphere is established as the unit of analysis, considering this to be the social subject’s nucleus of belonging to a community.
- Quality of life is analyzed as a process of conquests. Those processes are seen as a gradual upward trajectory allowing improvements to be attained in relation to one’s social vulnerability and conditions of precariousness, such as the deprivations in access to services and prejudices suffered by those living in extreme poverty.
- A favorable social context has coordinated offerings of facilities and other material, structural and human resources made available at a local level by public authorities through the prevailing programming and public policies. Such contexts entail the increased participation of families in community life.
- Seeking to construct and recreate the gradual, coordinated and collective processes with their complex respective factors that favor people’s attainment of autonomy within their family and community. This process of conquest leads toward improvements in quality of life.
- Conceiving of quality of life as a continuous process of reformulation. In this conception, there is no particular threshold to be reached, but instead a purpose in which objective and subjective gains and losses may be identified in association with life’s multiple facets. This process allows one to develop one’s potential and skills to overcome life’s hardships.
- Health promotion and quality of life are considered in the light of the supply and availability of access to resources that ensure comfort and satisfaction of essential (not minimum) prerequisites. Satisfactory access to such resources allows basic needs to be met, favors people’s human development in their families and communities, and facilitates the attainment of overall well-being.
- Quality of life is appreciated in association with local context and the singular reality of each nuclear family. A favorable quality of life is part of a complex landscape colored by several material, affective and symbolic aspects associated with one’s health conditions and environmental conditions. In this vision, quality of life is fertile land where one can develop one’s human potential despite life’s hardships.

#### Phase 3 – Quantitative

Application of the survey, in a representative sample of 336 heads of household (HH) with a profile of extreme poverty and target audience of the BFP, was used to investigate their views on local quality of life, coverage and access to public policies and health services, social assistance, education and the related effects on quality of life. The survey was applied to a systematic proportionate stratified sample of HHs in extreme poverty living in RN (*n* = 336), whose families were actively registered in the Single Registry Database (CadÚnico) of the Ministry of Social Development (MSD). Inclusion in the sample required the household to be registered in the CadÚnico database of families enrolled with a per capita income reflecting a situation of extreme poverty, as defined by the IBGE^1^ in 2014. The sample was calculated from a group of 2605 families with a profile reflecting extreme poverty. This represented approximately 18,000 individuals registered in CadÚnico with an inscription made between 2011 and 2014. The criteria for inclusion required household heads:

- to be at least 18 years old;
- to be residents in an urban area of RN for at least 6 months, and;
- to be conscious of the prevailing dynamics within the household, including information on every member of the household’s living conditions.

The finite population sampling formula was adopted for this calculation with a confidence level of 95% and a margin of error of 5%.

For data collection, the instrument was validated (i.e., tested in another field and optimized) previously. The final survey instrument comprised two parts and a total of 15 questions. The variables of the questions used in the survey were established with a basis in the research questions, as per the guidelines for survey methodology set out by Floyd, Fowler (2009) [34], Bradburn, Sudman, Wansink (2004) [35], and De Vaus (2004) [36]. The variables are detailed below:

- Socio-demographic data on families in extreme poverty: HH date of birth. Number of families residing in the household. HH birthplace. Age range of each of the family members disaggregated by sex. The household has a bathroom with water and piped sewerage. The household has electricity. The family owns a vehicle, motorcycle, bicycle or other means of transportation. The most common everyday type of trip performed by the family: home-work; home-school; home-health clinic. The time spent in the trip identified by the HH in minutes. The means of transportation used in the trip identified by the HH. The number of people in the family that: were formally employed; were informally employed; study in an educational institution; were retired; were unemployed; were on sick leave; were invalid; had special needs or physical handicaps. The family’s monthly income by type of income: formal employment; informal employment; retirement; stipend; government aid. Level of education: primary school; middle school; primary school complete; special primary school; secondary school incomplete; secondary school complete; undergraduate studies complete; graduate studies; none.
- Information on the quality of life of families in extreme poverty associated with BFP benefits and access to public services: Quality of life in RN and in the neighbourhood where the respondent resides: Likert Scale (Very good; Good; Regular; Bad; Very Bad). Bolsa Familia Program: If the respondent: is a beneficiary of the BFP; is no longer a beneficiary of the BFP; was never a beneficiary of the BFP. The amount of time that the respondent (has) received funds through the BFP: years and months. List of 24 types of public services (Table 2): if these public services exist in the respondent’s neighborhood or in neighboring (nearby) neighborhoods, or are absent. If the respondent does or does not use the 24 types of public services (Table 2). If these public services generally meet families’ basic needs: they do not meet any of the families’ needs; they meet few of their needs; they meet approximately half of their needs; they meet all their needs. Hierarchy ranking the importance of the main health problems in the community: List from 1 to 5 of the main health problems. Hierarchy ranking the importance of the main social determinants of health (SDH) prevailing in the community: List from 1 to 5 of the SDH (causes of the aforementioned health problems). Hierarchy ranking the importance of the main problems experienced in the territories with extreme poverty: List from 1 to 5 of the problems experienced in the territory. Hierarchy ranking the importance of the main suggestions for programming via investments in public policies to improve the quality of local life: List from 1 to 5 of the main intervention suggestions.

The data was collected between November 2014 and January 2015. The data was charted in Excel and processed with descriptive analysis.

#### Phase 4 – Qualitative and quantitative integration

The results of the qualitative and quantitative research were integrated, entailing a new phase of joint analysis. In accordance with the mixed methods framework [26], the researchers set out to identify possible perspectives with which findings could be drawn. In the spirit of the BCE theory, they aimed to obtain an in-depth interpretation of the reality experienced by families in extreme poverty in RN, their quality of life, access to public services and the BFP. The results describe the main ideas identified in this process, associating the views of M/Ps and of HHs on the matters of greatest relevance to the quality of life of families living in extreme poverty in RN.

In carrying out the study, the researchers complied with all ethical criteria outlined in the Resolution of the National Health Council No. 466, of 2012. Moreover, the project was approved by a research ethics committee through Opinion No. 188.866. All interviewees participated in the research by signing a free and informed consent form. Their anonymity was preserved throughout the study.

## Results

### Interviews with managers and professionals from public health, social assistance and education services

Of the 27 interviewees, 11 worked in the MHD, 11 in SAD, and 5 in the DE. Of these, 20 had undergraduate studies and 14 had post-graduate studies. Their average age was 35 years [25–63 years]. Sixteen M/Ps lived in other municipalities of the BHMA. Ten worked as civil servants under a statutory regime; 4 worked in commissioned positions; 17 worked on temporary contracts, 4 of which were in commissioned positions (repeated) (Table 1).

**Table 1:** Sociodemographic profile of managers and professionals of the public services in a municipality of the Belo Horizonte Metropolitan Area, Brazil

N	DEPARTMENT AND/OR UNIT	POSITION	EDUCATION	TIME IN POSITION
**MUNICIPAL HEALTH DEPARTMENT (MHD) PRIMARY CARE DEPARTMENT**				
**1**	Family Health Program Superintendency	Primary Care Coordination and Reference Authority	Nursing	1 year and 1 month
**2**	Food and Nutrition Program Superintendency	Food and Nutrition Surveillance	Nutrition	6 years
**3**	Family Health Unit A	Management, Technical Reference and FHS^a^ Assistance^a^	Nursing	4 years and 8 months
**4**	Family Health Unit B	Management, Technical Reference and FHS^a^ Assistance^a^	Nursing	4 years and 6 months
**5**	Family Health Unit C	Management, Technical Reference and FHS^a^ Assistance^a^	Nursing	3 years and 6 months
**6**	Family Health Unit D	Management, Technical Reference and FHS^a^ Assistance^a^	Nursing	1 years and 3 months
**7**	Family Health Team 1	Community Health Agent	High school studies	15 years
**8**	Family Health Team 2	Community Health Agent	High school studies	3 years and 6 months
**9**	Family Health Team 3	Community Health Agent	High school studies	11 years
**10**	Family Health Team 4	Community Health Agent	High school studies	16 years
**11**	Family Health Team 5	Community Health Agent	High school studies	10 years
**MUNICIPAL SOCIAL ASSISTANCE DEPARTMENT (MSAD) BASIC PROTECTION DEPARTMENT**				
**12**	Municipal Single Registry	Management	Studying Social Services	4 years
**13**	Municipal Bolsa Familia Program	Management and Central Technical Reference Authority	Administration	8 months
**14**	Regional Single Registry and Bolsa Familia Program	Management and Regional Technical Reference Authority	Social Services	1 years and 6 months
**15**	Municipal Food Bank	Management and Central Technical Reference Authority	Nutrition	1 years and 6 months
**16**	Productive Inclusion Superintendency	Management	Social Sciences	1 year
**17**	Regional Social Assistance Center of Reference 1	Management and Regional Technical Reference Authority 1	Social Services	4 years
**18**	Regional Social Assistance Center of Reference 2	Management and Regional Technical Reference Authority 2	Social Services	6 years
**19**	Local Social Assistance Center of Reference A	Coordination and Public Policy Analyst	Psychology	6 months
**20**	Local Social Assistance Center of Reference B	Coordination and Public Policy Analyst	Psychology	4 years and 6 months
**21**	Local Social Assistance Center of Reference C	Coordination and Public Policy Analyst	Psychology	6 months
**22**	Coexistence and Bond Strengthening Service	Coordination and Central Technical Reference Authority	Education	5 years
**MUNICIPAL DEPARTMENT OF EDUCATION (MDE) DEPARTMENT OF PROJECTS AND PUBLIC POLICY**				
**23**	Department of Projects and Public Policy	Management	Education	1 year
**24**	Superintendency and Inclusive Education	Management	Occupational Therapy	1 years and 2 months
**25**	Superintendency and Inclusive Education	Social worker	Social Services	14 years
**26**	Primary School	Supervisor of Education	Education	11 years
**27**	Primary School	Direction	Education	6 6 years

^a^*FHS* Family Health Strategy

A total of 15 analytical categories were identified regarding the views of M/Ps, which were grouped into four thematic groups. The M/Ps’ accounts, excerpts of which follow below, were attributed numbers and the abbreviations corresponding to the department for which they worked, as per Table 1. For example: “Account of the 7th interviewee from the Municipal Health Department” (7 MHD).

#### Quality of life of the population living in extreme poverty

The adverse living conditions in RN constituted a number of obstacles and impediments to attaining a good quality of life for its residents. This situation was all the more aggressive in connection to meeting the basic needs of families living in extreme poverty, as told by the M/Ps.

Living in extreme poverty was tantamount to the imprisonment of such families in this context. Their social, community, and family bonds were progressively fragmented, culminating in damages to their health:

*“These people suffer. Sadness weighs on their lives!” (25 DE)*

*“Their quality of life is hard; the fathers go to work at 5 a.m. and get back at 10 p.m. The children are in the projects all day long... It’s quite a hard life!” (15 SAD)*

*“The family is fragmented, they have no sense of coexistence. They live together, but they aren't a family...” (27 DE)*

*“There are 10,542 people living in extreme poverty according to CadÚnico, but the IBGE Census reflects a smaller number of people living in poverty... These people aren't aware of the opportunities out there. They’re passive individuals, prisoners in a cocoon!” (13 SAD)*

*“Life in these conditions ends up entailing fragmented family and social bonds, which are passed on from parents to their children. In this way, the adversity that they face on a day-to-day basis becomes a common, shared reality for these families, between parents and their children, and together with the whole community. So, there are no prospects for a different life for them to strive for!...” (1 MHD).*

#### Reflections on the inefficiency of public policies and prejudice to quality of life and health for families in extreme poverty

The M/Ps affirmed that coverage and provision of public services in RN were both precarious. For them, this accentuated certain objective (material) and subjective (psychosocial) detriments to the families while further removing the prospect of a better life, generating a sense of imprisonment in the cycle of poverty. This would be a consequence of inefficiency on the part of public authorities to ensure access to the essential public services required to promote a life with dignity:

*“The population is linked to basic health units that are references, but they're very far away. They don’t have schools or facilities. Most of them live on lands they do not formally own... They’re neighborhoods without urban infrastructure, with precarious wastewater networks and overflowing...” (8 MHD).*

*“There are neighborhoods with heavy poverty that don't have a single registered institution, that have no coverage. There are many alcohol users. There’s a need for work, but there are no jobs...” (15 SAD).*

*“Depression is the most common illness as their children do drugs and the mothers just suffer. Most of these mothers have no profession, low education and low self-esteem... They've got the basics: rice, pasta, beans, pork rind... Their health becomes a snowball... Lots of hypertension among men and hypertensive crises among women. The problem is not only their health, but a serious social problem that unleashes hypertension, diabetes; it increases medication without any effects. Antidepressants continue on the rise... But the problem is this person and their family’s context!” (4 MHD)*

Receipt of social aid, like registration in the Single Registry, was a voluntary responsibility of families. There were not enough teams from the public social assistance, health and education services to seek out the entire population in extreme poverty. This exacerbated the invisibility of that population and the lack of adhesion to the measures designed for them. This was the case of the BFP and the holistic care provided through the Family Health Strategy (FHS):

*“The service only manages to work with a few of the families in this situation...” (13 SAD)*

#### Detriment to the quality of life and health of families in extreme poverty with negative consequences for their autonomy

There are aggregated chain reactions between the various factors that affect the quality of life of those living in extreme poverty in material (objective) and immaterial (subjective) terms. Namely, they consist in detriments to the living conditions of such families, a loss of autonomy and of their drive to face the struggle and/or search for improvements, whether in the personal, family and/or collective spheres.

*“There are no spots in daycares for working mothers. It becomes a chain of problems linked to lacking access that keeps families in their state of poverty.” (15 SAD).*

*“The quality of life in poverty is very low, poor, in very precarious conditions, without access to health, water supply and sanitation services, without housing or access to education. These are families with difficulties accessing these things. That's complicated. They live in far-away places, remote, in precarious conditions, without any means to get around, go to school, a health center; they can't make that contact!” (1 MHD)*

#### Precarious quality of life, imprisoned in an inter-generational cycle of extreme poverty

The M/Ps affirmed that families in extreme poverty become passive and conform to their local reality. This, they reported, was due to the context marked by so many adverse factors and a denial of their universal rights. The social inequalities that they suffer on a day-to-day basis imprison families in the territories where they live, generating spatial segregation and social exclusion in a reality characterized by a lack of prospects and “isolation” from society.

*“There are invisible families that have no access, that are nowhere, that exist only where they live... They don't exist in the eyes of the authorities”. (15 SAD).*

*“They’re like a very trodden path, where nothing grows anymore... They’ve already been trodden on by the government, since they're lacking everything, lacking every basic thing... They feel excluded, poorly treated and, what’s worse, there is a complete lack of community organization to fight for local improvements...” (19 SAD).*

### Survey with household heads of families in extreme poverty

The general characteristics of the survey were:

- 78% female sex;
- average age: 41 years and 2 months (standard deviation ±14 years and 8 months)
- born in other municipalities of Minas Gerais or States: 93.5%
- 58% completed primary education;
- average number of residents per household: 3.9.

The residents with an income up to one-third of minimum wage represented 56.9% of the sampled population. Those with income between one-third and two-thirds of minimum wage represented 31.1%. Those receiving social assistance such as income support through BFP represented 44.6%, while another 23.2% had previously received social assistance through the BFP. Another 29.2% of household heads had registered to receive assistance through the BFP but never received income support. The families that received income support through the BFP did so for an average period of 4.3 years. Regarding job market inclusion, 50.6% had family members of working age, working without workers’ rights normally granted via an employment contract (informal employment); 55.8% had 1 or more members of working age, unemployed, unsuccessfully looking for work; 40.2% had an unemployed member.

Those with one or more members studying in the formal education system represented 66.7%; those with one retiree per household represented 17.3% and with two retirees, 6.3%. Among the houses visited, 77.1% had a bathroom with water and piped sewage; 10.1% had piped water and a septic tank; 11% had only piped water; and 99.7% had electricity.

There were no means of transport in 49% of households; 12.5% used bicycles; 7.8% used a motorcycle; and 30.5% used a car. Regarding the most significant types of travel performed by families on a day-to-day basis, 53.7% cited the home to work commute; 25.4% home to school; and 12.5% home to the health center.

For work, the most used means of transport was public transport, which was used by 32.1%. Of these, 55.1% spent approximately 120 min or more commuting daily from home to work (round trip). From home to school, 45.1% spent up to 30 min a day (round trip), and 45.1% spent 30 to 90 min going to school and back.

From home to the health center, 26.7% spent less than 30 min, round trip; 33.3% spent 30 to 60 min; 26.2% from 60 to 90 min; 11.9% from 120 to 180 min.

Regarding quality of life in neighborhoods where families lived, 1.8% referred to it as very good; 20.2% good; 36.6% regular; 22.9% bad; and 18.5% very bad.

Regarding coverage of public health and education services, respectively, 86.9 and 86.3% considered them present in the territories where they lived and/or nearby neighborhoods. Coverage of social assistance services, especially the Social Assistance Reference Centers (CRAS), were considered as present by 18.8% of respondents, as per Table 2.

**Table 2:** Coverage, access and use of available public services in territories with poverty by families living in a municipality in the Belo Horizonte Metropolitan Area, Brazil – 2015

TYPES OF PUBLIC SERVICES AVAILABLE TO MEET BASIC NEEDS	RF^**a**^ % OF COVERAGE AND ACCESS TO PUBLIC SERVICES IN TERRITORIES OF RESIDENCE			RF^**a**^ % OF PUBLIC SERVICE USE If available in the Neighborhoods where families resided or Nearby regions	
	NEIGHBOURHOOD	NEARBY REGION	ABSENT	YES	NO
Basic Education Schools (primary and secondary school)	86.9	11.6	9.5	55.0	35.1
Daycare	48.2	13.7	38.0	9.2	53.0
Higher education and/or Professional Vocational Training	5.1	7.4	87.2	2.4	10.7
Health Clinic, Health Center, Family Health Care Unit	86.3	8.9	3.6	86.9	8.6
Social Assistance Reference Centers (CRAS) or Social Support Service	18.8	24.1	57.0	18.2	24.7
Public Safety Service, Police Station and/or Policing Service	25.3	21.7	52.4	15.0	32.0
Public or Collective Transportation	87.5	7.7	4.0	90.0	6.0
Waste collection	93.5	1.5	4.5	92.0	3.3
Parks, Squares and/or Green Spaces	18.8	11.9	68.2	14.9	16.7
Access to Internet	48.2	4.2	47.0	32.0	20.2
Food Bank, Food Supply Service, Basic Food Basket Distribution Service	2.1	3.9	93.8	2.0	4.5
Cultural Activities: Theater, Dance, Visual arts, Music...	0.5	3.0	92.0	2.4	5.1
Sports activities, Courts, Outdoor Exercise	25.9	12.5	61.3	11.3	27.1
Access to Public Telephones or Phone Booths	62.8	6.6	30.4	21.0	48.0
Public Library or Library Open to the Public	22.3	7.1	69.9	16.3	70.0
Distribution Service for Popular Medicine, Popular Pharmacies or Clinics	73.5	11.9	14.0	73.2	12.5
Water Supply System	94.6	–	4.8	93.0	–
Sewage Network	84.5	–	15.2	77.0	7.0
Electricity Distribution	97.3	–	2.4	95.0	1.2
Paved Roads, Asphalt, Sidewalks	67.3	–	31.6	–	–

^a^RF %: Relative Frequency

Regarding the provision of available public services, 53.3% stated that “*they did not meet any or few of the everyday needs of families in the region*”; and 36.9% stated that they met “*half of their needs*”. Only 8.6% considered that public services in the region met “*all their needs*”.

They also mentioned the main health problems in the community, the main social determinants of health, and the main problems in territories of extreme poverty, prioritized by degrees of importance. These are presented in Table 3.

**Table 3:** Main health problems, social determinants of health and territorial problems of families in extreme poverty living in a municipality of the Belo Horizonte Metropolitan Area, Brazil-2015

	MAIN HEALTH PROBLEMS IN THE COMMUNITY	MAIN SOCIAL DETERMINANTS OF HEALTH	MAIN PROBLEMS IN TERRITORIES OF EXTREME POVERTY
**1st**	Alcohol and drug abuse and dependence (48.2%)	Alcohol and drugs (68.8%)	Lack of public security (66.7%)
**2nd**	Hypertension (45.5%)	Lack of good health care (60.7%)	Unpaved roads (64%)
**3rd**	Asthma, Bronchitis (35.1%)	Lacking income, work, employment (37.5%)	Lacking work and employment (47%)
**4th**	Dengue (36%)	Lack of support and social exclusion (38.4%)	Lacking sport and leisure (58.6%)
**5th**	Flu and cold (45.5%)	Family and social stress (32.1%)	Absence of daycares (47.3%)

Moreover, they pointed out the main problems, in order of priority, to be addressed in territories with extreme poverty. Addressing these would help to achieve improvements in the quality of life of families through investments in public policies 1) health (40.5%); 2) education (37.8%); 3) employment, paid work (44.6%); 4) culture and leisure (35.5%); and 5) security, policing in the residence’s neighborhood (45%).

## Discussion

This study applied a mixed methods approach to gather qualitative and quantitative data on the following themes: extreme poverty, universal public policies (coverage of and access to services for health care, education, social assistance, social protection and urban infrastructure) in association with targeted public policy programming (BFP), quality of life, basic capability development (BCE), the right to life and health, citizenship and well-being. The findings suggest that being trapped in extreme poverty is the reflex of a chain of integrated factors that constitute processes of social inequality. These processes are reflections of the inequality inherent to public policy planning and implementation in the territories with extreme poverty in RN. This situation highlights the reality of families living in extreme poverty in RN without any prospect of developing their basic capabilities. Those families are hindered from attaining well-being due to the general inadequacy of the local public policies, where living conditions, quality of life and access to public services such as health care are precarious.

Situations of spatial segregation and social exclusion—such as those experienced by families of RN living in extreme poverty, as verified in this study—reproduce the cycle of imprisonment in situations of extreme poverty. These situations demonstrate the chain of social determinants of poverty in conjunction with social determinants of health. The residents’ situations entailed detrimental impacts on their quality of life, their health potential and illnesses. The families found themselves trapped in a cycle of social exclusion facing alcoholism, drug addiction, depression, unemployment, and a great loss of autonomy as concerns their efforts to fight for local improvements. The effects of poverty in association with precarious lifestyles on health require further studies. Indeed, few studies exist that address health in situations of poverty [37].

Situations of extreme poverty exist in large urban centers and recur in many cities in Brazil, Latin America and around the world. It is known that contexts of socioeconomic crises affect families living in poverty in ways that further aggravate situations of unemployment, stigma related to unemployment and social exclusion. In turn, chronic stress is prone to worsen and physical and psychological illness, such as depression [38], further intensify as observed in a large number of this study’s results (*“Depression is the most common illness as their children do drugs and the mothers just suffer”; “*Alcohol and drug abuse and dependence” was identified by 48.2% of HHs as the main social determinant of health). In this regard, a study focusing on social support for mothers with mental health problems and children in situations of vulnerability observed that social support represents a protective factor that contributes to getting families out of poverty [39].

The BCE framework [9] made it possible to clarify various challenges faced by families in extreme poverty in this study regarding gaps in coverage and access to public services. Such gaps require mediation by public authorities to ensure residents enjoy their citizenship rights, attain their full potential state of health and develop their capabilities with autonomy. It was found that the principle of social justice, applied in the different public policies in RN, is simply “utilitarian” and does not adopt the principle of equity proposed by Amartya Sen through BCE [9]. To do so, these families’ geographical, spatial, social, economic and educational limitations would have to be respected. Access to public services is required to compensate for their seclusion in extreme poverty. Indeed, Amartya Sen contends that social exclusion and lacking capabilities are leading reasons for poverty. The capabilities approach is offered as a way for people to attain well-being and freedom of choice, as it allows them the greatest possibility to develop their own individual capabilities. In this sense, access to public services is key to ensure people’s well-being. Furthermore, social security systems constitute a set of instruments mediated by public authorities with the ability to reduce inequality and vulnerability [18], as highlighted in this study’s findings.

With the number of people living in cities surpassing previous levels, the urbanization of poverty is an increasingly common process. The increase in urban poverty (90%) has mainly taken place in lesser developed regions. Poverty is a special problem and the main challenge in reaching families living in poverty is related to the complex spatial dynamics of poverty. In particular, urban poverty presents significant challenges associated with spatial connections, aspirations, opportunities and access to public transportation [40]. In Latin America, social inequalities and the proportion of the population in poverty are both high. Of the 30.2% who live below the poverty line, mainly in large urban cores, 30% do not have access to health care due to financial limitations and 21% do not seek care due to geographical barriers [41].

Human capital is critical for economic growth. Investments in health and education are widely recognized as mediating factors for national development in all developed and developing countries. One study has highlighted that public investments in health and education reduced poverty in 12 member countries of the OPEC [15]. Other studies have also verified benefits to economic growth via external assistance provided to countries with high indices of poverty. In such cases, investments in areas such as education, health, treated water, basic sanitation, improvements to public transportation, energy, communications and urban infrastructure led to increased income for the population living in poverty [42–44].

The fragile employment conditions of professionals in the health, education and social assistance sectors and their short-lived assignments to teams with temporary contracts, as indicated by the findings, contribute to shortcomings in these services. Their teams are often incomplete and unable to adequately carry out the relevant programming and actively search for families in extreme poverty. Providing health professionals with long-term assignments is fundamental to enable them to maintain a bond with families and to ensure adequate monitoring and universal coverage in the scope of Primary Health Care (PHC) and FHS, such as through social assistance and education services [45–47]. Household heads identified access to health care and education as the main public policy programming required to improve the quality of life in the territories where they lived (40.5 and 37.8%, respectively).

Moreover, the findings echo Sen’s observations [9] on the great problem of imprisonment in the cycle of poverty. He claims that this situation can generate an adaptation process wherein the excluded person feels so diminished in terms of their personal aspirations that their desires turn stagnant or even self-degrading. Their life vision becomes so twisted that they perceive their precarious quality of life as “satisfactory”, thus limiting themselves to their entrapment in poverty. Such people’s counterfactual preferences must be considered to analyze extreme poverty, inquiring about the life that individuals would choose to live if they were not subject to such circumstances. The M/Ps of the various public services pointed out that “*These people suffer. Sadness weighs on their lives!*” and “*They’re like a very trodden path, where nothing grows anymore...*” due to the precarious and aggressive circumstances in which they live owing to lacking access. Yet, 20.2% of heads of households cited their quality of life as good, 36.6% as regular, 22.9% as bad, and 18.5% as very bad. Sen [9] highlights that individuals living in extreme poverty reproduce the misfortunes linked to social exclusion in their own lives as if they were somehow acceptable. In doing so, such individuals end up increasingly trapped in a cycle with ever decreasing self-esteem and rejected personal potential. The negative attributes of a life in poverty are commonly associated with a process that affects “other people”, since poverty entails marginalization and stigmatization, which contributes to keeping individuals in poverty trapped in the cycle [48].

Sen points out that this twisted vision of one’s own quality of life can be called a “mistake” as it allows a contradiction in society to emerge regarding equal rights in a simplistic way. Mainly, such perceptions divert focus away from the central issue of social inequality, which public policies are meant to address. Addressing social inequalities would require legitimate, coherent processes to manage public policies backed by integrated measures and intersectoral strategies targeting the most vulnerable groups to increase their possibilities of achieving other, as yet denied, life prospects [9]. It is important to identify the nature and causes of discrimination, social exclusion, and inequality in all societies. Doing so can contribute to more appropriate planning and implementation of public policies with a mandate to tackle poverty [49].

Yet, the significant gap in research is noteworthy—at the national and international levels—involving the themes of governance, poverty, and quality of life. There is a dearth of information on plausible and applicable ways to facilitate collaborative decision-making in cooperative work and community networks with the support of priority measures [50] that help to cope with the social determinants of health and poverty [34, 45, 51]. More in-depth research is needed from scholars of poverty to identify the causes of this phenomenon, of inequality and of deprivation [48]. Moreover, it is essential to obtain a better understanding and additional academic support to build human and social capital in association with measures to tackle vulnerabilities and community poverty [18].

Regarding the dissemination of information on public policies with a mandate to tackle poverty in Brazil, Social Assistance Reference Centers (CRAS) are the services responsible for identifying such families and promoting their inclusion in the BFP. However, the number of CRAS in RN is insufficient (18.8%, Table 2) which makes it difficult to assist these families with their needs. This situation is made worse by the fact that health and education services do not make up for the CRAS’s insufficiencies. These services also do not manage to provide sufficient coverage in various territories where families live in extreme poverty, as their teams are sometimes short-staffed.

In a national survey on the use of health services in Brazil, it was found that the more health care facilities there were for the population to overcome barriers in the use of the Single Health System (SUS), the more use was made of PHC and preventive services. This survey helped to conclude that the expansion of the SUS, and especially the FHS, would be very relevant for the most vulnerable and lacking groups in terms of access to health [52]. Indeed, the same was validated in the present study. Therefore, it is essential to fortify public services in Brazil. Despite all the country’s historical advances in public services, substantial improvements are still required—mainly in terms of supply to the most vulnerable groups. Improvements must advance constitutional guarantees for coverage and universal access in all regions. Priority should be given to the poorest people, who are the most historically unassisted groups [45, 50–52].

Regarding the role of the SDGs in tackling poverty, SDG1 aims to “eradicate extreme poverty” everywhere by 2030 [53]. Moreover, SDG3 reiterates the importance of equality and the need for economic, social and environmental development to incorporate this principle to ensure “healthy lives and promoting well-being for all at all ages”. Thus, health inequality monitoring is to be valued as it generates evidence that can and should be used to inform equality-oriented policies, programs and practices that align with intersectoral nature of the SDGs. In this regard, implementing equity-oriented change may require action across health and non-health sectors and involve multiple stakeholders [16].

When the World Bank put the SDGs into practice, it adopted a modified poverty line from $1.25 to $1.90 (US). With this change, the number of poor people in Latin America increased from 19 million to 37 million [54]. In this regard, Brazil stands out as, since 2015, it began to experience a strong socioeconomic crisis with an intense resurgence of extreme poverty. In 2018, about 4.5 million people in Brazil entered extreme poverty, contributing to the level of inequality in the country reaching a record level; the population in extreme poverty accounted for 13.5 million people [5]. However, for some authors, Brazil is not a poor country but is instead marked by the determinants of poverty. This would derive from the association of many processes ingrained into structures of perversely unequal income distribution, a historically well-established fixture in the country. The culmination of which is the absence of opportunities for economic and social inclusion [55]. Based on the BCE framework, this study contends that investments in health care, education and social assistance are a way to minimize the adverse attributes of poverty in Brazil and in other countries. Similarly, a study on country members of the OPEC identifies the value of human capital in reducing poverty, highlighting that investments in health care and education contributed to attaining a higher standard of living as well as economic growth [15].

In this study, extreme poverty proved to be the reflection of a cascade effect that trapped and restrained the capabilities of families in RN. To tackle this, policies targeting poverty in Brazil would require priority, such as the BFP. Especially in RN, measures are needed that aim to increase coverage and universal access to health and education. Such measures should reduce social inequalities in association with other intersectoral public policies related to transportation, housing, leisure and employment, as per the findings of several other studies [47, 53, 56]. It is important to perform analyses and to assess the trends of management processes, prioritizing social determinants of health in association with the principle of intersectorality [56, 57].

Programming must be carried out that aims to ensure coverage and universal access to health, education and social assistance, in conjunction with advances in the BFP. Such programs would provide necessary strength to social protection mechanisms in Brazil, promoting improved quality of life for families in extreme poverty, and attaining the SDGs: 1 - poverty eradication and 3 - health and wellbeing.

Regardless of the country’s economic crisis, it is necessary to value social rights together with economic growth, prioritizing action that targets the most vulnerable territories [50, 53, 56–59]. The effects of economic crises in developing countries must be mitigated, prioritizing sustainable public funding of public services to the benefit of public welfare and progressively reducing potential health risks [60]. Greater investments are needed in social security, based on the BCE approach, in order to maximize human capital development (basic capabilities and personal potential), ensure adequate well-being, and fulfil the rights to health, education, social security and urban infrastructure. Such investments must be accompanied by equitable intersectoral public policies that target the areas of territories where community life takes place [18, 45, 51].

Among the limitations of this study, there is the fact that the characteristics of the phenomenon “quality of life in extreme poverty” are clarified without developing tools to address the problems identified regarding actual management practices. This is a challenge for other studies addressing strategies for intersectoral public policies. By working with indicators that address the quality of life of people living in situations of poverty, such studies could provide previews of ways to improve the quality of life of such families. However, the application of the BCE framework’s premises in this study provided understanding of the chain of social determinants of poverty in conjunction with the social determinants of health. It also clarified the implicit consequences of processes of inequality in the distribution of public policies.

Greater reflections are required to further clarify the reality experienced by these groups. In this way, society could start to believe in and support principles of social justice that are less “utilitarian” and supportive of the status quo regarding social inequalities, and rather more directed to social justice and equity (BCE) [9] as a precept for the right to life.

In many parts of the world, threats to the value of solidarity constitute the basis of crises that impact on social protection systems. But it is in developing countries, where these systems are even more fragile and less inclusive, where such threats can further intensify social inequalities and poverty [61]. Therefore, in light of the objective living conditions of citizens in their territories, it is necessary to rethink and coordinate the interfaces of public services with care for social protection in Brazil and focus on constructing citizenship rights for all [62].

There is no single, universally applicable method to assess the multidimensional reality experienced by families in extreme poverty. The notion of poverty must be understood with an appreciation for what constitutes a favorable space in which to develop one’s capabilities. Such spaces must enable individuals to advance through gradual processes to attain progressive improvements in their quality of life and well-being [63]. Identifying the different dimensions of poverty is a necessary priority to handle the diverse, legitimate programming designed to tackle poverty better. The basic capability approach, geared toward developing human and social capital, draws attention to the matter of poverty and the associated--and diverse--intersectoral policy approaches. Programming should empower subjects and develop their human capital. The absence of basic, accessible public services for families in poverty is a condition restricted to those living in poverty. Public authorities at a federal, state and local level have a mandate to supply public services that meet all individuals’ basic needs. Moreover, their mandate includes the function of ensuring social functionality by effectively promoting access to services via facilities that ensure individuals enjoy an adequate standard of living with adequate well-being and an active role in social life [40].

Finally, with the BCE [9] framework in mind, it is concluded that equality should not be measured in simplistic “egalitarian” terms. These only reflect the distribution of income and the availability of material goods and primary public services that are sometimes inaccessible. Instead, equality should be the quest for an “equal capability to function”. That would, in fact, allow everyone to desire and make efforts to attain a satisfactory quality of life with adequate well-being.

## Conclusions

The BCE framework was applied to analyze the living conditions of families in extreme poverty in RN. It allowed the researchers to obtain an in-depth understanding of the objective and subjective factors at play in the chain of social and health determinants of the inter-generational cycle of extreme poverty. Individuals’ imprisonment in this cycle was a consequence of their deprivation as relates to accessing material goods (such as income, productive inclusion and employment) in association with essential public services (such as health care, education, social assistance, public transportation, among others). Such services are considered essential to meet those families’ basic everyday needs. Deprivations led to losses affecting the residents’ health potential, including illnesses such as depression, alcoholism and drug addiction; the two latter illnesses were identified as the main health problems in the community and the main social determinants of health. The available public services in the territories with extreme poverty in RN were insufficient to meet the basic needs of resident families. Indeed, inadequacies in services impeded them from developing their potential and human capital, from attaining well-being, and from enjoying citizens’ rights. The subjects in this study suggested that priority be given to programming and investments in health care to achieve improvements in their quality of life.

The social determinants of poverty and health act in conjunction, eroding the health potential and human capital of the families in extreme poverty and hindering their choices and opportunities to improve those subjects’ quality of life. These determinants, in turn, must be addressed jointly. For this, intersectoral public policies must be designed and implemented with a basis in the BCE framework to foment the development of basic capabilities and human capital and the attainment of well-being. In this regard, public policies must underscore the value of social security and intersectoral design and execution, both at the individual and collective levels. Furthermore, public policies should be supported by assessments of the reality experienced by families in extreme poverty that take into account the multiple dimensions of poverty (i.e. objective ones, such as material goods, and subjective ones related to psychosocial perspectives). In this way, the implementation of public policy programming will gain legitimacy and generate favorable outcomes for those subjects’ quality of life.

## Data Availability

All the data for this study is available and it can be accessed at the Instituto de Pesquisas René Rachou with autors.

## Footnotes

1 IBGE: Brazilian Institute of Geography and Statistics.
